# Cytoplasmic dynein heavy chain: the servant of many masters

**DOI:** 10.1016/j.tins.2013.08.001

**Published:** 2013-11

**Authors:** Giampietro Schiavo, Linda Greensmith, Majid Hafezparast, Elizabeth M.C. Fisher

**Affiliations:** 1 Sobell Department of Motor Neuroscience and Movement Disorders, Institute of Neurology, National Hospital for Neurology and Neurosurgery, University College London, Queen Square, London WC1N 3BG, UK; 2 Molecular NeuroPathobiology, Cancer Research UK London Research Institute, 44 Lincoln's Inn Fields, London WC2A 3LY, UK; 3 MRC Centre for Neuromuscular Disease, Institute of Neurology, National Hospital for Neurology and Neurosurgery, University College London, Queen Square, London WC1N 3BG, UK; 4 School of Life Sciences, University of Sussex, Falmer, Brighton BN1 9QG, UK; 5 Department of Neurodegenerative Disease, Institute of Neurology, National Hospital for Neurology and Neurosurgery, University College London, Queen Square, London WC1N 3BG, UK

**Keywords:** amyotrophic lateral sclerosis, axonal transport, Cramping 1, Legs at odd angles, motor neurons, neurodegeneration, Sprawling

## Abstract

Cytoplasmic dynein is the main retrograde motor in all eukaryotic cells. This complex comprises different subunits assembled on a cytoplasmic dynein heavy chain 1 (DYNC1H1) dimer. Cytoplasmic dynein is particularly important for neurons because it carries essential signals and organelles from distal sites to the cell body. In the past decade, several mouse models have helped to dissect the numerous functions of DYNC1H1. Additionally, several *DYNC1H1* mutations have recently been found in human patients that give rise to a broad spectrum of developmental and midlife-onset disorders. Here, we discuss the effects of mutations of mouse and human DYNC1H1 and how these studies are giving us new insight into the many critical roles DYNC1H1 plays in the nervous system.

## Highlights

• The cytoplasmic dynein complex is the main retrograde motor in all eukaryotic cells.
• This complex is built around a dimer of cytoplasmic dynein heavy chains (DYNC1H1).
• Mouse DYNC1H1 mutants have sensory defects, but motor defects have been controversial.
• Now human DYNC1H1 mutations with sensory, motor, and cognitive deficits are being found.
• The study of these mutations will give us new insight into DYNC1H1 function in the nervous system.

## Introduction

**Figure 1:**
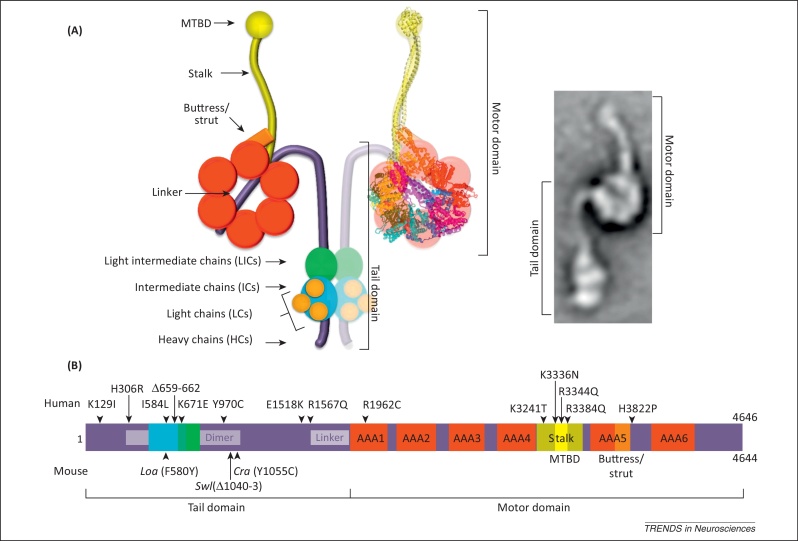
The cytoplasmic dynein complex. (**A**) Diagram of the cytoplasmic dynein motor complex including the heavy chain (HC) dimer and its associated subunits. A model of the motor domain [5] built from yeast cytoplasmic dynein (PDB ID 4AKG) and the mouse microtubule-binding domain (MTBD) (PDB ID 3ERR) assembled by Dr A.P. Carter has been overlapped with the schematic of the dynein HC in its apo or post-power stroke form [5,93,94]. Adapted, with permission, from The Company of Biologists (*J. Cell Sci*. 126, 705–713; [4]). The electron micrograph of an isolated molecule of monomeric dynein from *Chlamydomonas reinhardtii* flagella in its pre-power stroke form is shown for comparison on the right. Adapted with permission from Macmillan Publishers (*Nature* 421, 715–718; [93]). Conformational changes driven by ATP hydrolysis in the motor domain, which alter the relative position of the stem and the tail/linker, are hypothesised to lead to the power stroke and progression on microtubules [5,94]. The HCs (in dark violet) contain the six AAA ATPase domains (in red), the stalk region, which includes the MTBD (in dark yellow and yellow, respectively), the buttress (in orange), and the linker region. HCs are associated with light intermediate chains (LICs) (in green), intermediate chains (ICs) (in cyan), and light chains (LCs) (in light yellow). (**B**) Domain composition of the cytoplasmic dynein HC. In addition to the functional domains shown in (A), this scheme displays the homodimerisation region and linker (in white). The positions on the dynein HC of the three mouse mutations (*Loa*, Legs at odd angles; *Cra*, Cramping 1; *Swl*, Sprawling; bottom) and the human mutations discussed in this review (top) are indicated.

Cytoplasmic dynein 1 is a large (∼1.5 MDa), multisubunit motor complex (Figure 1A) that moves towards the minus end of microtubules in eukaryotic cells [1]. It belongs together with the axonemal dyneins and cytoplasmic dynein 2 to the dynein superfamily. Axonemal dyneins are responsible for the movement of cilia and flagella, whereas cytoplasmic dynein 2 has a role in intraflagellar transport and is required for cilia and flagella assembly [1].

The core of the cytoplasmic dynein 1 complex is the heavy chain (DYNC1H1) dimer (Figure 1A). Each heavy chain is enormous – a half-megadalton protein – and, perhaps unsurprisingly, serves multiple purposes. Towards the N terminal it has a long tail domain with binding sites for other structural and regulatory components of the dynein complex and docking sites for cargoes including adaptor proteins (Figure 1B and Table 1); at the C terminal, DYNC1H1 folds into a daisy-like structure comprising six ATPase domains associated with diverse cellular activities (AAA+) and a microtubule-interacting stalk region [2,3] (Figure 1A,B). This motor domain drives the entire complex and its cargoes along microtubules [2,4], although it is not completely understood how ATP hydrolysis is coupled to force generation or even the total number of ATP molecules bound to DYNC1H1 at any given time [4–6].

DYNC1H1 is highly conserved and is an essential protein in higher eukaryotes because the dynein complex has housekeeping functions in all cells, including orientation of the mitotic spindle, nuclear positioning, Golgi maintenance, and endosomal dynamics [7–9]. In the nervous system, the complex takes on additional roles specific to neurons; driven by the heavy-chain motor, it transports cargoes within dendrites and is the sole motor supporting retrograde transport in axons – carrying signalling complexes that affect gene expression, development, and regeneration, misfolded proteins, and organelles from the synapse back to the cell body. Interestingly, the analysis of motor function in axons revealed that motor proteins, including cytoplasmic dynein, strictly rely for axonal transport on the local synthesis of ATP by glycolytic enzymes, which are bound to transported organelles [10,11], and not on mitochondrial ATP.

In mammals, new insight into DYNC1H1 function came from characterising a set of dynein heavy chain mouse mutations. Now mutations in humans are also being found. Both mouse and human data show that the dynein heavy chain is essential for normal function of the nervous system and even single conservative amino acid substitutions in this > 500-kDa protein can result in neurological abnormalities.

## An allelic series of DYNC1H1 mutants for understanding the role of cytoplasmic dynein in the nervous system

### Mouse Dync1h1 mutant strains

A phenotype arising from a single point mutation gives a snapshot of protein function. By working with multiple mutations of one protein, an ‘allelic series’, we gain a much richer picture – particularly helpful when trying to dissect the function of a large protein like the cytoplasmic dynein heavy chain.

Although fly and worm laboratories have worked at the molecular level with multiple mutant alleles of the dynein heavy chain for many years, the first mammalian DYNC1H1 mutation came in 1998 with a knockout mouse (Table 2) [12]. In mouse and human, DYNC1H1 appears to be a single isoform encoded by 78 exons [13]. Heterozygous knockout mice had no reported phenotype, but homozygous nulls died early in gestation (<8.5 days) with Golgi apparatus, endosome, and lysosome abnormalities, underlining that DYNC1H1 is an essential protein with housekeeping roles.

Insight into the nervous system function of *Dync1h1* came with three further mutants: Legs at odd angles (*Dync1h1*^*Loa*^), Cramping 1 (*Dync1h1*^*Cra1*^), and Sprawling (*Dync1h1*^*Swl*^) mice [14,15] (Table 2 and Figure 1B). *Dync1h1*^*Loa*^ mice present a single point mutation from a phenylalanine to a tyrosine (F580Y) in the dynein tail domain. Surprisingly, the addition of a single hydroxyl group onto the 532-kDa protein is enough to give rise to a dominant phenotype [15]. This residue lies within the binding site for dynein intermediate chains (ICs) [16] (Figure 1B) and the homodimerisation region [16,17]. *Dync1h1*^*Cra1*^ mice have instead a single point mutation at the opposite end of the homodimerisation domain (Y1055C) (Figure 1B) that causes a neurological phenotype overlapping that of *Dync1h1*^*Loa*^ [15–17] (Table 2). *Dync1h1*^*Loa*^ and *Dync1h1*^*Cra1*^ were generated by independent chemical mutagenesis experiments (Box 1); a radiation-induced mutant, *Dync1h1*^*Swl*^, has a small deletion (G1040–T1043delinsA) in the proximity of the *Dync1h1*^*Cra1*^ mutation (Table 2 and Figure 1B) and also produces a dominant phenotype that primarily affects the nervous system [14,18,19].

Homozygous *Dync1h1*^*Loa*^ and *Dync1h1*^*Cra1*^ mice die by 1 day after birth. This could potentially be due to a large reduction in spinal cord motor neurons, limiting their ability to feed [15]. By contrast, *Dync1h1*^*Swl*^ homozygotes die at the late implantation–early gastrulation stage [14,18,19].

### Human DYNC1H1 mutations

In 2010, the first human *DYNC1H1* mutation (*DYNC1H1*^*H3822P*^; Figure 1B and Table 3) was found in an individual with developmental delay, hypotonia, and brain malformations [20]. These phenotypic manifestations are of great interest because cytoplasmic dynein is essential for neuronal migration. A second mutation (*DYNC1H1*^*E1518K*^) has also been found to cause severe intellectual disability and other features [20,21]. Recently, Poirer and colleagues reported seven novel *de novo* mutations (two of them overlapping) and a small deletion, in addition to a familial mutation, in individuals with malformations of cortical development (MCD)^22^. MCD is characterised by alterations of the cerebral cortex and is often associated with severe intellectual disability. The mutations causing MCD are located in different domains of *DYNC1H1*, but several of them (*DYNC1H1*^*K3241T*^, *DYNC1H1*^*K3336N*^, *DYNC1H1*^*R3344Q*^, *DYNC1H1*^*R3384Q*^) cluster in the stalk region and on the surface of its microtubule-binding domain (MTBD) (Figure 1B) and have been shown to weaken binding of the dynein complex to microtubules [22]. The remaining mutations lie in the first AAA ATPase domain (AAA1) (*DYNC1H1*^*R1962C*^), in an area proximal to the linker region (*DYNC1H1*^*R1567Q*^) and at the N terminus (*DYNC1H1*^*K129I*^) of the tail domain, whereas the deletion (*DYNC1H1*^*Δ659-662*^) was found within the binding sites for ICs and Light Intermediate Chains (LICs) [16] (Figure 1B and Table 3). Although the N terminus of the tail domain is not involved in *in vitro* motility or in the binding of structural and regulatory components, the dramatic effect of the *DYNC1H1*^*K129I*^ mutation (Table 3), together with mutagenesis analyses in *Aspergillus* [23], suggest that this region of DYNC1H1 is critical for cytoplasmic dynein functions *in vivo*, possibly for the coordination of force production and movement under heavy load [23].

*DYNC1H1* mutations have also now been reported in motor neuropathies, which may be accompanied by cognitive impairment. The first such family presented with the dominant hereditary distal motor neuropathy Charcot–Marie–Tooth (CMT) type 2, with delayed motor milestones and/or abnormal gait. Affected individuals have early-onset lower-limb weakness and wasting caused by a mutation, *DYNC1H1*^*H306R*^, within the homodimerisation domain of the dynein heavy chain [24] (Figure 1B and Table 3). However, an independent family with an identical mutation manifests symptoms of spinal muscular atrophy (SMA) [25]. Other *DYNC1H1* mutations have been found by exome sequencing in families with a similar SMA presentation [26]. The key clinical features in individuals with three different mutations in the tail domain (*DYNC1H1*^*I584L*^, *DYNC1H1*^*K671E*^, *DYNC1H1*^*Y970C*^) were congenital or very early-onset weakness in the proximal legs and other areas and a static or mildly progressive disease course. Clinical findings support a diagnosis of motor neuron disease without sensory involvement [26,27]. Interestingly, *DYNC1H1*^*K671E*^ and the *DYNC1H1*^*Δ659-662*^ deletion are only 10 residues apart, yet their main clinical outcome, MCD associated with spastic tetraplegia, and motor neuron disease, respectively, are dramatically different.

The human and mouse mutations described so far are all autosomal-dominant disorders with high penetrance; it is unknown whether the phenotypes arise from gain of function or hemizygous loss of function or, perhaps more likely in such a large protein, a combination of both. There is, however, evidence that the *Dync1h1*^*Loa*^ mutation results in reduced affinity between cytoplasmic dynein and the p150 subunit of dynactin (DCTN1), a binding partner of dynein (Table 1) [28] and in impaired motor processivity [28,29], although it is unclear whether this is the sole cause of the phenotype.

## Consequences of DYNC1H1 mutations in the nervous system

### Locomotor and motor system deficits

*Dync1h1*^*Loa*,^ ^*Cra1*,^ ^*Swl*^ heterozygous mouse mutants have gait deficits that manifest by approximately 1 month of age (Table 2). They limb-clench when held by the tail and have grip-strength deficits, which occur as early as 1 week after birth in *Dync1h1*^*+/Swl*^ mice [14,15]. *Dync1h1*^*+/Cra1*^ mice have early-onset reduction in grip strength, which does not progress [30] or mildly [31] progresses with age. Forelimb strength and movement are preserved in *Dync1h1*^*+/Loa*^ mice [32].

Interestingly, all of the human *DYNC1H1* mutation cases have gait abnormalities ranging from spastic tetraplegia, inability to walk at all, and ‘waddling gait’ to mild distal limb weakness; *pes cavus* or other foot deformities are often a feature, indicating an underlying neuromuscular disorder [20,21,24–27,33] (Table 3). The striking similarity between the locomotor phenotypes observed in humans and mice suggest that DYNC1H1 plays crucial roles in the physiological control of the motor system in both species.

The first description of heterozygous *Dync1h1*^*+/Loa*^ and *Dync1h1*^*+/Cra1*^ mice reported a progressive loss of motor neurons with a predominance of large type I fibres in specific muscles [15]. However, later reports found no loss of alpha motor neurons in *Dync1h1*^*+/Loa*^ [14,34] or *Dync1h1*^*+/Cra1*^ [30,35] mice. Although these contradictory results are complicated by possible methodological differences (e.g., how different laboratories count large alpha motor neurons), they may also give insights for unravelling the effect of genetic factors in dynein function, because one possible source of variation is the genetic background of the mice examined in different papers. Our original report [15] described motor neuron loss in *Dync1h1*^*+/Loa*^ mice with a mixed genetic background that included at least C3H, PTP, 101, and C57BL/6, whereas subsequent studies were all on congenic C57BL/6 [14,34]. It is therefore possible that genetic background plays a modifying role on this phenotype.

This would not be the first case in which genetic background affects phenotype severity. In an established mouse model of amyotrophic lateral sclerosis (ALS) (*SOD1*^*G93A*^), the C57BL/6 background extends life compared with a C3H background [36]. Extrapolating to the *Dync1h1* mutants, it is possible that alleles in the mixed genetic background used in the original study enhanced motor neuron loss whereas the C57BL/6 background was protective.

However, genetic effects are not an explanation of the discrepancies found in *Dync1h1*^*+/Cra1*^ and *Dync1h1*^*+/Swl*^ mice [14,15,35], because these studies were performed on mice of the same (C3H) or very similar (C3H/101) background. Curiously, neuromuscular junctions (NMJs) have been found to be normal in *Dync1h1*^*+/Cra1*^ mice on a congenic C3H background [35], whereas on a mixed genetic background that includes C3H, NMJs were reported as abnormal [30].

As expected, embryos bearing homozygous mutations in *Dync1h1* (e.g., *Dync1h1*^*Loa/Loa*^ and *Dync1h1*^*Cra1/Cra1*^) display augmented phenotypes with severe motor neuron loss, although at present it is unknown whether these are developmental or neurodegenerative defects.

How do these results help in deciphering the clinical manifestations of human patients bearing mutations in *DYNC1H1*? Motor neuropathy in human patients displays different degrees of severity and includes distal wasting and weakness with reduced or absent reflexes and chronic distal denervation [27]. Distal motor neuron loss has been suggested in *DYNC1H1*^*H306R*^, *DYNC1H1*^*I584L*^, *DYNC1H1*^*K671E*^, and *DYNC1H1*^*Y970C*^ patients [24–26,33], whereas the *DYNC1H1*^*E1518K*^ individual has hypertonia and did not learn to walk [20,21]. Two interesting observations arise from the human data that strengthen the correspondence with the results obtained in the mouse studies and may relate to the modifier effects of genetic background found with the mouse mutations. First, the human *DYNC1H1*^*I584L*^ mutation lies almost on top of the *Dync1h1*^*Loa*^ mutation and is also very conservative (Ile to Leu). The functional characterisation of this human mutation indicates loss or dysfunction of motor neurons, akin to the original observation in the *Dync1h1*^*+/Loa*^ mice [15]. Henceforth, the *Dync1h1*^*+/Loa*^ mouse may represent an ideal system to model the pathophysiological effects of the *DYNC1H1*^*I584L*^ human mutation. Second, *DYNC1H1*^*H306R*^ manifests with distinct symptoms in different human pedigrees. In one (UK pedigree), patients display a predominant axonal CMT2B disease [24], whereas in the other (Japanese pedigree) they present with SMA with lower extremity predominance [25], potentially demonstrating the strong effects of genetic background also in the human cases.

### Sensory system defects

In addition to motor system deficits, mutations in the dynein heavy chain also cause impairments in other areas of the nervous systems, including sensory neurons. An overt sensory phenotype is found in *Dync1h1*^*+/Swl*^ mice, which have early-onset sensory neuropathy probably due to degeneration during late embryonic development [14,18] (Table 2). These mice have proprioceptive and nociceptive sensory neuron loss compared with wild types [14]. Sensory nerve conduction velocities are reduced and the mice have moderate sensory neuropathy arising from the proprioceptive defect [14]. Similarly, *Dync1h1*^*+/Loa*^ mice have progressive loss of sensory axons from at least 1.5 months after birth, mostly from proprioceptive nerves [34]. Branching and elongation of primarily sensory limb nerves is impaired in E13.5 and E14.5 *Dync1h1*^*Loa*^ homozygotes and heterozygotes [15]. *Dync1h1*^*+/Cra1*^ mice have milder loss of sensory axons than other *Dync1h1* mutants and, perhaps surprisingly, no difference in proprioceptive neuron number or morphology compared with wild types at 6 months of age [30].

All three mutant strains of mice were identified because of hind-limb clenching and low-based gait (Table 2). In *Dync1h1*^*+/Loa*^ and *Dync1h1*^*+/Swl*^, these features may be due to proprioceptive defects. However, in *Dync1h1*^*+/Cra1*^ they may arise from dysfunction of the NMJ rather than motor or sensory neurodegeneration, which fits with findings from *Drosophila* and other model organisms in which dynein disruption results in NMJ abnormalities [30].

This sensory phenotype is also found in human patients with *DYNC1H1* mutations, but with important differences that may be explained at least in part by modifier genes associated with a different genetic background. Individuals bearing the *DYNC1H1*^*H306R*^ mutation range from having mild reduction of proprioception to deficits in all sensory modalities in the UK pedigree [24], but normal sensory nerve conduction and no obvious sensory abnormalities in the Japanese pedigree [25]. Surprisingly, several human patients with *DYNC1H1* mutations have no obvious sensory loss [22,26,33] (Table 3), raising the possibility that this is due to a permissive genetic background in these patients. An alternative explanation is that the human sensory system is more robust than in rodents and is protected from the deleterious effects of *DYNC1H1* mutation by compensatory mechanisms. In this regard, the resulting sensory phenotype may be too subtle to be clinically relevant and detected by current protocols.

### Abnormalities in brain morphology and function

*Dync1h1* mutations affect brain function at multiple levels. Brain imaging showed striatal atrophy and lateral ventricle enlargement in *Dync1h1*^*+/Cra1*^ mice, which also had altered dopamine signalling. However, neuronal loss was found only in the substantia nigra [31]. In humans, Perry syndrome, an atypical Parkinson-like disease, can be caused by point mutations in the p150 subunit of dynactin [37] (Table 1). Furthermore, cytoplasmic dynein directly binds huntingtin (Htt) and huntingtin-associated protein 1 (HAP1), two proteins involved in the physiology of the dopaminergic system, via dynactin [38] (Table 1).

Correct cortical development also requires cytoplasmic dynein. MCD has been detected in several cases with *DYNC1H1* mutations [21,22] and is often associated with microcephaly, pachygyria (presence of pathological thick convolutions of the cerebral cortex), polymicrogyria (excessive number of small convolutions of the cortex), and other central nervous system (CNS) malformations. Patients often display early-onset or focal epilepsy and mild-to-severe intellectual disability (Table 3). Interestingly, two cases (*DYNC1H1*^*K3336N*^ and *DYNC1H1*^*R3384Q*^) display both pachygyria and polymicrogyria patterns, which are normally regarded as distinct clinical entities and have been found associated only in patients with WDR62-related brain abnormalities [39].

These malformations probably arise because the cytoplasmic dynein complex is important for neuronal migration [40]. This may also explain the abnormal migration of facial motor neuron cell bodies to the hindbrain found in E10.5 *Dync1h1*^*Loa/Loa*^ embryos [15]. This striking phenotype was not observed for cranial and spinal nerves in the neural tube, suggesting that the susceptibility of distinct neuronal subtypes to the same *Dync1h1* mutations varies within the same genetic background. This possibility is strengthened by the finding that alpha rather than gamma trigeminal motor neurons or proprioceptive neurons are affected in *Dync1h1*^*+/Loa*^ mice, showing abnormal dendrites, aberrant mitochondria, and other defects consistent with altered trafficking of organelles [41]. Uncovering the mechanistic basis of this differential sensitivity will greatly further our understanding of the factors determining type-specific neuronal cell death as a response to generic insults or mutations of ubiquitously expressed proteins, a major unsolved problem in basic and clinical neuroscience.

**Figure 2:**
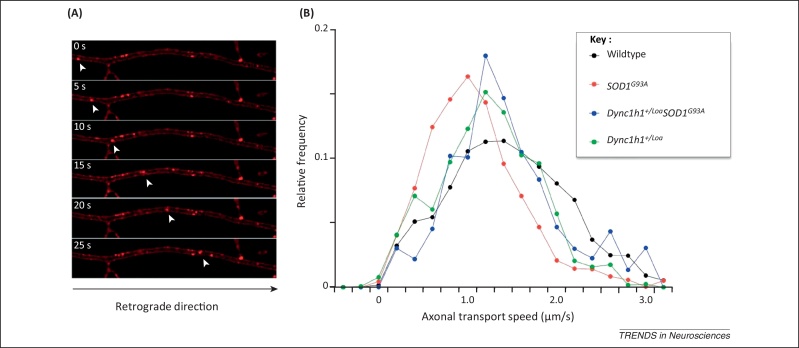
Quantitative analysis of axonal retrograde transport by intravital microscopy. (**A**) Axonal retrograde transport of signalling endosomes containing a fluorescently labelled atoxic fragment of tetanus neurotoxin was monitored in single axons in the intact sciatic nerve by time-lapse confocal microscopy and shown as a time series [44]. (**B**) The deficit in axonal retrograde transport observed in early symptomatic *SOD1*^*G93A*^ transgenic mice (74 ± 1.7 days; in red) is almost completely rescued by the *Dync1h1*^*Loa*^ allele in *Dync1h1*^*+/Loa*^*SOD1*^*G93A*^ double-mutant mice (in blue). The speed distribution profile displayed by *Dync1h1*^*+/Loa*^*SOD1*^*G93A*^ mice overlaps with that observed in *Dync1h1*^*+/Loa*^ animals (in green) and is slightly shifted towards lower speed values compared with wild type mice (in black) of similar age.

Based on the importance of cytoplasmic dynein in microtubule-dependent movement [42], the effects of *Dync1h1* mutations on axonal transport were tested. Motor neuron cultures from *Dync1h1*^*Loa/Loa*^ but not *Dync1h1*^*+/Loa*^ embryos show a highly significant reduction in axonal retrograde transport [15,43]. The lack of an overt phenotype in heterozygous neurons is unexpected because mutant DYNC1H1 acts as a dimer [15,28]. The DYNC1H1^*Loa*^ protein displays impaired run-lengths on microtubules [29] and the human DYNC1H1^I584L^ protein has decreased binding to microtubules in the presence of ATP [26]. Both mutations have a similar biochemical profile, which is interesting given their proximity and highly conserved nature. The lack of an axonal transport phenotype in cultured *Dync1h1*^*+/Loa*^ embryonic motor neurons was confirmed by assessing the retrograde transport of signalling endosomes in the sciatic nerve of adult *Dync1h1*^*+/Loa*^ mice by intravital microscopy [44]. This technique allows the quantitative real-time assessment of the retrograde movement of transport organelles in the intact sciatic nerve of living mice and revealed only a minor drop in high transport speed in *Dync1h1*^*+/Loa*^ mice (Figure 2), suggesting that only a subset of the specific functions of dynein is affected in these mice, causing a mild phenotype compatible with life.

## Mutations in dynein heavy chain and neurodegenerative diseases

Although human *DYNC1H1* mutations have been described causing CMT2 neuropathy (*DYNC1H1*^*H306R*^) [24], SMA (*DYNC1H1*^*H306R*^) [25], (*DYNC1H1*^*I584L*,^ ^*K671E*,^ ^*Y970C*^) [26], and severe cognitive/neuronal migration deficits (*DYNC1H1*^*E1518K*,^ ^*H3822P*^) [20,21], to date some of the most interesting results for dissecting disease mechanisms come from the mouse mutants. In this section, we focus on a subset of breeding experiments testing the genetic interactions of *Dync1h1* mutations with known genes important for neurodegenerative diseases.

### ALS

ALS is a neurodegenerative disease that usually manifests in midlife and results in progressive loss of upper and lower motor neurons, leading to paralysis and death within 3–5 years of diagnosis. Around 10–20% of ALS is an inherited autosomal-dominant disease and of these familial cases, about 10% are due to mutations in the superoxide dismutase 1 (*SOD1*) gene. Mice carrying human mutant SOD1 transgenes model ALS [36,45] and develop deficits including defective axonal retrograde transport (for example, see [43,44,46]).

*SOD1*^*G93A*^ transgenic ALS mice were crossed to *Dync1h1*^*+/Loa*^ mice and double-mutant progeny (i.e., *SOD1*^*G93A*^*Dync1h1*^*Loa*^) surprisingly lived 28% longer than their *SOD1*^*G93A*^ transgenic littermates, with a significant increase in survival of spinal cord motor neurons at a 120-day late-disease time point compared with *SOD1*^*G93A*^ littermates [43] (Table 4). Curiously, double mutants had an increased rate of axonal retrograde transport in embryonic motor neuron cultures compared with their single-mutant and wild type littermates [43]. Interestingly, the *Dync1h1*^*Loa*^ allele is able to rescue almost completely the axonal transport deficits observed in early symptomatic *SOD1*^*G93A*^ mice, as shown by intravital microscopy (Figure 2). This restoration of axonal retrograde transport may determine the amelioration of the ALS disease phenotype observed in *Dync1h1*^*Loa*^*SOD1*^*G93A*^ double mutants, including significant life extension (see below).

This phenomenon was unexpected and therefore similar experiments were performed by different laboratories by crossing *Dync1h1*^*+/Loa*^ mice to various mutant *SOD1* strains. Although the increase in lifespan is reproducible (21% life extension was found in an identical cross [14]), changing the genetic background of *SOD1*^*G93A*^ mice to C57BL/6 reduced this increase to only 9% [34] (Table 4). This effect was confirmed using a different *Dync1h1* mutation (*Dync1h1*^*+/Cra1*^). Indeed,,a 14% increase in time to humane end point was found in *Dync1h1*^*+/Cra1*^*SOD1*^*G93A*^ double mutants compared with *SOD1*^*G93A*^ littermates [47]. Further support for a functional interaction between *SOD1* and the dynein motor is provided by work using a transgenic mouse with chronically impaired cytoplasmic dynein and dynactin function because of neuronal expression of Bicaudal D2 N terminus (BICD2^GFP-N^) [48]. BICD2 is a conserved motor-adaptor protein that is involved in dynein-mediated transport by linking the dynein motor complex to various cargoes (Table 1). It plays a major role in organelle trafficking in various cells, including neurons, and its mutation causes autosomal-dominant SMA and hereditary spastic paraplegia (HSP) [49–51]. Double mutants of *BICD2*^*GFP-N*^ with ‘low-copy’ *SOD1*^*G93A*^ mice also show an increased lifespan (+14%), further supporting a genetic interaction between *SOD1* and cytoplasmic dynein and suggesting that the expression level of the *SOD1*^*G93A*^ transgene is not likely to play a role in this phenomenon.

What is the molecular mechanism at the basis of the amelioration of the ALS phenotype by *Dync1h1* mutations? Expression of SOD1^G93A^ in primary motor neurons alters the cellular localisation of cytoplasmic dynein [52]. As a consequence, *SOD1*^*G93A*^ mice have axonal transport defects and a change of cargoes in the axon from survival signals to stress/death signals [53]. Thus, inhibiting a specific subset of retrograde organelles may help delay the activation of cell stress pathways. This could help motor neurons to survive SOD1^G93A^ toxicity [53] and may determine the rescue of axonal retrograde transport deficits observed in *Dync1h1*^*Loa*^*SOD1*^*G93A*^ double mutants by intravital microscopy (Figure 2). Additionally, differences in the expression of the main anterograde motor kinesin 1 have also been noted in *Dync1h1*^*+/Cra1*^*SOD1*^*G93A*^ mice [54].

Although the presence of a direct interaction between DYNC1H1 and SOD1 remains controversial [55,56], *Dync1h1*^*Loa*^*SOD1*^*G93A*^ mice have significant reductions in mutant SOD1 protein in the mitochondrial matrix [57] that determine amelioration of mitochondrial respiration and restoration of membrane potential in embryonic motor neurons. Therefore, these neurons are more resistant to the toxic effects of mutant SOD1 on mitochondria [57]. This phenotype is surprising given that mouse fibroblasts derived from *Dync1h1*^*+/Cra1*^ and *Dync1h1*^*Cra/Cra1*^ embryos display profound alterations of mitochondrial morphology and that *Dync1h1*^*+/Cra1*^ mice develop, hyperinsulinemia, hyperglycaemia, and progressive mitochondrial dysfunction [58]. Similar mitochondrial abnormalities have also been reported in fibroblasts isolated from SMA patients carrying the *DYNC1H1*^*I584L*^ and *DYNC1H1*^*K671E*^ mutations [58].

Tau redistribution has also been called on to explain the increased lifespan found in double-mutant animals. *Dync1h1*^*+/Cra1*^*SOD1*^*G93A*^ mice display some restoration of tau isoform ratios compared with single-mutant animals, although the main effects were in the cortex and cerebellum rather than the spinal cord [59]. *Dync1h1*^*Cra1*^*SOD1*^*G93A*^ mice also show increased systemic expression of insulin-like growth factor 1 (IGF-1), which may contribute to the amelioration of the *SOD1*^*G93A*^ disease phenotype [60]. Indeed, viral delivery of IGF-1 to the CNS has been shown to delay motor neuron death in SOD1^G93A^ mice [61], although the efficacy of IGF-1-based therapy in ALS patients remains controversial [62]. An improved disease outcome may also result from reduced excitotoxicity caused by loss of glutamatergic proprioceptive sensory neurons [34]. However, this seems unlikely because the *Dync1h1*^*Swl*^ mutation, which causes a greater loss of such neurons, does not rescue the mutant *SOD1*^*G93A*^ phenotype [14] (see below).

Despite these intriguing results, no significant increase in lifespan was observed when the *Dync1h1*^*+/Loa*^ mouse was crossed to two other *SOD1*-ALS models: dismutase-active *SOD1*^*G37R*^ and dismutase-inactive *SOD1*^*G85R*^ transgenic mice [34]. Similarly, a comparable *Dync1h1*^*+/Swl*^ cross with *SOD1*^*G93A*^ mice showed no effect of this dynein mutation on lifespan [14] (Table 4). Despite active research in this area, the molecular basis for these discrepancies is presently unknown, although the slower progression, lower penetrance, and severity of the *SOD1*^*G37R*^ and *SOD1*^*G85R*^ mutant phenotypes may explain some of the experimental differences found using these strains.

### Huntington's disease (HT) and other neurological diseases

Functional interactions with other genes implicated in human neurodegenerative diseases, in addition to ALS, have also been inferred. Treatment of cells expressing mutant Htt or alpha-synuclein, which are mutated in HD and Parkinson's disease (PD), respectively, with a cytoplasmic dynein inhibitor caused inhibition of the clearance of both aggregate-prone proteins by autophagy [63]. When *Dync1h1*^*+/Loa*^ mice were crossed to an HD model, tremor onset and severity were greatly enhanced by the dynein mutation in the double-mutant progeny, which had significantly shorter lifespans [63]. This was thought to arise from the deleterious effects of the *Dync1h1*^*Loa*^ mutation on autophagy, which relies on cytoplasmic dynein for the intracellular transport and fusion of autophagosomes with lysosomes [63]. Interestingly, Htt and HAP1 help to control vesicle transport by interacting with the dynein–dynactin complex [38] and mutant Htt adversely affects dynein transport of brain-derived neurotrophic factor (BDNF) [64,65]. Crucially, Htt protein levels are decreased in dynein-mutant mice [64]. Interestingly, defects in brown and white adipose tissues were found in *Dync1h1*^*+/Loa*^ and *Dync1h1*^*+/Cra1*^ mice, reminiscent of those seen in human HD patients, potentially because of the functional role of dynein in lipid droplet trafficking [66].

Enhancement of the severity of the disease phenotype was also observed in other animal models of neurodegeneration crossed with *Dync1h1*^*+/Loa*^ mice. Mutations in the enzyme glycyl-tRNA synthetase (*GARS*) cause motor and sensory axon loss in humans due to dose-dependent gain of function and give rise to clinical phenotypes that range from forms of CMT neuropathy to severe infantile SMA. A mouse with a missense mutation in the *Gars* gene that has locomotor and sensory deficits was crossed to *Dync1h1*^*+/Loa*^ mice and, as expected, the double mutants were more severely affected than either parent [67]. However, it is presently unclear whether this increase in severity is linked to a defect in the known role of GARS in translation, amino acid mischarging, or a still unknown mechanism [68].

Several pathogens and virulence factors exploit cytoplasmic dynein and other molecular motors to reach their site of action or replication, harness the biosynthetic machinery towards their replication compartment, or leave their host and spread [69] to other cells or tissues. In agreement with this view, vesicles containing mammalian prion protein (PrP^C^) have been shown to engage both cytoplasmic dynein and kinesin 1 for their intracellular movement [70] and clinical prion disease correlates with impaired axonal transport in motor neurons [71]. However, no differences in prion disease incubation times were found in *Dync1h1*^*+/Loa*^ and wild type littermates inoculated intraperitoneally and intracerebrally with mouse-adapted scrapie protein [72]. This result suggests that, although PrP^C^ undergoes dynein-mediated intracellular trafficking, PrP^Sc^ toxicity relies on alternative transport mechanisms independent of this molecular motor. This conclusion is in agreement with recent evidence showing that the plasma membrane is a primary site for prion conversion [73,74] and that the transfer of PrP^Sc^ among adjacent cells could occur via tunnelling nanotubes [75].

## Concluding remarks and future perspectives

The cytoplasmic dynein heavy chain is involved in housekeeping and neuron-specific cellular processes. The first allelic series of *Dync1h1* mutations in mice provided important insight into the function of this motor complex, but at the same time highlighted some phenotyping differences between investigating laboratories that remain incompletely understood. Crucially, several mutations now found in human *DYNC1H1* are enriching the picture and clearly show pivotal roles for this motor complex in nervous system function. Disruption of DYNC1H1 results in developmental and degenerative defects, which may be modulated by genetic background. It is highly likely that more human and mouse mutations will be found, which will provide us with a better understanding of the roles played by this very large protein. Many intriguing questions remain (Box 2), such as why the known mutations have overlapping phenotypes but remain distinct from each other. This could be partly explained by potential disruption of binding by different cargoes and adaptors [42], for example, and we note the strong correlation in phenotype between the mouse *Dync1h1*^*Loa*^ and the human *DYNC1H1*^*I584L*^ mutations. Newly identified variants in regions of *Dync1h1* that are still poorly understood, such as the buttress, the linker region, and the C-terminal end of the molecule (Figure 1B), will provide us with novel information on how these domains contribute to the force-generating process and determine the step size of this molecular motor. Similarly, we have only a partial understanding of the coordination of the six AAA ATPase subunits. Novel mutations in this region of *DYNC1H1*, such as the recently discovered *DYNC1H1*^*R1962C*^ in AAA1, will greatly help us to dissect the precise mechanism of ATP hydrolysis and how chemical energy is translated into mechanical force.

With respect to neurodegeneration, defects in axonal retrograde transport, which is mainly driven by cytoplasmic dynein, have been reported in several neurodegenerative diseases [42,46,76] and cytoplasmic dynein trafficking is involved in processes that are often aberrant, including stress granule formation and trophic factor signalling. The intriguing finding of extended lifespan in *Dync1h1*^*+/Loa*^*SOD1*^*G93A*^ double-mutant mice may inspire new avenues of research into novel potential drugs modulating axonal retrograde transport rate. Sadly, even a 10% increase in lifespan in humans with ALS would currently be a triumph. Thus, the interplay of mutant SOD1 and DYNC1H1 deserves further investigation as well as the reason why the lifespan-enhancing effect of *Dync1h1*^*+/Loa*^ has been found, to date, only with a specific human SOD1 mutation.

With respect to development, a recent study has shown that DYNC1H1 is of fundamental importance for cell-size sensing, especially in neurons, and indeed there are defects in neuronal length in *Dync1h1*^*+/Loa*^ mice [77]. The nature of the signals transported by cytoplasmic dynein regulating cell size and the pathways responsible for this process are presently unknown. However, their discovery holds promises for the exploitation of this machinery to sustain axonal regeneration and homeostasis in pathologies in which axonal integrity is compromised, such as CMT2 and HSP [78].

We have confined this review to data obtained from studies in mice and humans, but several results from other model organisms also support the key role of cytoplasmic dynein in neuronal physiology. These include studies in *Drosophila*, where the creation of an allelic series of cytoplasmic dynein heavy-chain (*cDhc64C*) mutants provided an early demonstration that disruption of *cDhc64C* (and the dynactin orthologue p150^Glued^) causes bidirectional disruption of axonal transport [79], thus suggesting that retrograde and anterograde transport are tightly coordinated. More recently, the zebrafish *dync1h1 cannonball* mutant has disrupted photoreceptor morphogenesis, revealing multiple roles for the protein in retinal development [80]. Importantly, the general principles learned by studying cytoplasmic dynein in metazoans can be transferred to lower organisms. The *Dync1h1*^*Loa*^ mutation has been recreated in *Neurospora*, allowing further dissection of the molecular effects including alterations in dynein localisation and impaired speed of vesicle transport [81]. Future studies using protocols allowing the reconstitution of human cytoplasmic dynein from recombinant subunits [82] will allow the *in vitro* assembly of cytoplasmic dynein complexes containing specific heavy-chain mutants, such as those described in mouse and human animal models. These mutant complexes may then be tested in *in vitro* motility assays, allowing us to uncover the secrets of this fascinating molecular motor and its complex network of interacting proteins and regulators. Integration of *in vitro* studies addressing how cytoplasmic dynein works at the molecular level with future genetic investigations in mice and humans will be key to understanding how cytoplasmic dynein juggles such a range of functions and its so many physiological masters.

## Figures and Tables

**Table 1:** Cytoplasmic dynein-interacting proteins^a^

Protein	Type of binding	Site/subunit	Refs
LIS1	Direct	DYNC1H1 (AAA3/AAA4 junction) DYNC1H1 (AAA4 arginine finger)	[83]
NudE	Indirect	Intermediate chain Light chain – LC8	[84]
Dynactin	Indirect	Intermediate chain	[85]
Snapin	Indirect	Intermediate chain	[86,87]
Htt	Indirect	Intermediate chain	[88]
HAP1	Indirect	Dynactin	[88]
BICD1 BICD2	Indirect Indirect	Dynactin Intermediate chain	[89,90] [91]

a The table shows some of the interactors with the cytoplasmic dynein motor complex involved in CNS development and homeostasis.

**Table 2:** Mouse mutations in the cytoplasmic dynein heavy chain gene

Approved allele name	Allele	Phenotype of heterozygotes	Refs
*Mouse Dync1h1*^*tm1Noh*^	Knockout, created by gene targeting of the first exon.	Heterozygotes reported normal. Nulls die by embryonic day (E)8.5 with Golgi and other abnormalities.	[12]
*Mouse Dync1h1*^*Loa*^	Point mutation resulting in missense change F580Y in homodimerisation and dynein intermediate chain-binding site; created by chemical mutagenesis.	Motor, sensory, and other abnormalities. Loss of 50% motor neurons in E18.5 embryos. By 13 weeks, muscle spindles are reduced by 86% in hind limbs. Homozygotes dead by 1 day after birth.	[14,15]
*Mouse Dync1h1*^*Swl*^	9-bp deletion resulting in loss of three amino acids from 1040–1043 (GIVT to A); created by radiation mutagenesis.	Sensory early-onset neuropathy, with reduction of 88% of muscle spindles in hind-limb muscles compared with wild type. Homozygotes die *in utero* before E8.5.	[14,19]
*Mouse Dync1h1*^*Cra1*^	Point mutation resulting in missense change Y1055C in homodimerisation domain, created by chemical mutagenesis.	Motor, sensory, and other abnormalities. Loss of 20% of motor neurons in E18.5 embryos. Heterozygotes display progressive mitochondrial dysfunction in muscle, hyperinsulinemia, and hyperglycaemia, progressing to glucose intolerance with age. Homozygotes dead by 1 day after birth.	[15,58]

**Table 3:** Human mutations in the cytoplasmic dynein heavy chain gene

Approved allele name	Allele	Phenotype of heterozygotes	Refs
*Human DYNC1H1*^*K129I*^	*De novo* point mutation resulting in missense change K129I at the N terminus of the tail domain.	Normocephaly. Pathological thick convolutions of the posterior cerebral cortex (posterior pachygyria) and severe intellectual disability. Late-onset epilepsy.	[22]
*Human DYNC1H1*^*H306R*^	Point mutation resulting in missense change H306R in homodimerisation domain.	Early-onset, slowly progressive distal lower-limb weakness and wasting (similar to CMT2 neuropathy) or SMA with lower-extremity predominance. Learning difficulties in some individuals.	[24,25]
*Human DYNC1H1*^*I584L*^	Point mutation resulting in missense change I584L in homodimerisation and dynein intermediate chain-binding site.	SMA with lower-extremity predominance. Normal upper-extremity strength.	[26,33]
*Human DYNC1H1*^*Δ659-662*^	*De novo* 12-bp deletion resulting in loss of four amino acids from 659–662 in homodimerisation and dynein intermediate chain-binding site.	Microcephaly associated with posterior pachygyria. Early-onset epilepsy and spastic tetraplegia.	[22]
*Human DYNC1H1*^*K671E*^	Point mutation resulting in missense change K671E in homodimerisation and dynein intermediate chain-binding site.	Early-onset, slowly progressive distal lower-limb weakness and wasting. May have a ‘waddling’ gait. No known sensory involvement.	[26]
*Human DYNC1H1*^*Y970C*^	Point mutation resulting in missense change Y970C in homodimerisation domain.	Significant motor delay, no known sensory involvement, mild cognitive impairment.	[26]
*Human DYNC1H1*^*E1518K*^	*De novo* point mutation resulting in missense change E1518K.	Severe mental retardation, unable to walk or talk, hypertonia and club feet; untested reflexes. Epilepsy. Cortical malformation. Lack of sensory data.	[20,21]
*Human DYNC1H1*^*R1567Q*^	*De novo* point mutation resulting in missense change R1567Q.	Normocephaly. Excessive number of small convolutions of the frontal cortex (frontal polymicrogyria) and severe intellectual disability. Foot deformities.	[22]
*Human DYNC1H1*^*R1962C*^	*De novo* point mutation resulting in missense change R1962C in AAA1.	Normocephaly. Posterior pachygyria with severe intellectual disability and awkwardness. Transient focal epilepsy at early age.	[22]
*Human DYNC1H1*^*K3241T*^	Familial point mutation resulting in missense change K3241T in the stalk domain.	Normocephaly. Posterior pachygyria with mild or absent intellectual disability and variable awkwardness. Focal epilepsy.	[22]
*Human DYNC1H1*^*K3336N*^	*De novo* point mutation resulting in missense change K3336N in the stalk domain (microtubule-binding domain [MTBD]).	Microcephaly associated with posterior pachygyria, frontal polymicrogyria, and other CNS malformations. Early-onset epilepsy. Spastic tetraplegia with foot deformities.	[22]
*Human DYNC1H1*^*R3344Q*^	Point mutation resulting in missense change R3344Q in the stalk domain (MTBD). This *de novo* mutation was reported in two unrelated individuals.	Mild microcephaly associated with posterior pachygyria with moderate intellectual disability and awkwardness. Focal epilepsy.	[22]
*Human DYNC1H1*^*R3384Q*^	*De novo* point mutation resulting in missense change R3384Q in the stalk domain (MTBD).	Microcephaly associated with posterior pachygyria, frontal polymicrogyria and other CNS malformations. Early-onset epilepsy. Spastic tetraplegia with foot deformities.	[22]
*Human DYNC1H1*^*H3822P*^	*De novo* point mutation resulting in missense change H3822P in a linker region between AAA5 and AAA6 within the motor domain.	Hypotonia, moderately severe mental retardation, broad-based, waddling gait, reduced reflexes. Bilateral deficient gyration of the frontal lobes. Lack of sensory data.	[20,21]

**Table 4:** Genetic interactions between *SOD1* and the cytoplasmic dynein complex^a^

*Dync1h1* mutant mouse parent	SOD1-ALS mutant mouse parent	Effect on lifespan of double mutants compared with SOD1-ALS littermates (time to humane end point shown for SOD1-ALS double mutants compared with their SOD1-ALS littermates)	Refs
*Dync1h1*^*+/Loa*^ on C57BL/6 congenic background	*SOD1*^*G93A*^ transgenic on hybrid SJL, C57BL/6 background	28% significant increase in lifespan (160 days, *n* = 18, compared with 125 days, *n* = 20)	[43]
*Dync1h1*^*+/Loa*^ on C57BL/6 congenic background	*SOD1*^*G93A*^ transgenic on hybrid SJL, C57BL/6 background	21% significant increase in lifespan (156 days, *n* = 12, compared with 129 days, *n* = 16)	[14]
*Dync1h1*^*+/Loa*^ on C57BL/6 congenic background	*SOD1*^*G93A*^ transgenic on C57BL/6 congenic background	9% significant increase in lifespan (165 days, *n* = 9, compared with 152 days, *n* = 9)	[34]
*Dync1h1*^*+/Cra1*^ on C3H background	*SOD1*^*G93A*^ transgenic on congenic C57BL/6 background	14% significant increase in lifespan (167 days, compared with 147 days, *n* = 14/15)	[47]
*Dync1h1*^*+/Swl*^ on C3H/101 background	*SOD1*^*G93A*^ transgenic on hybrid SJL, C57BL/6 background	No significant differences in lifespan (124 days double mutants, *n* = 15, compared with 122 days single mutants, *n* = 15)	[14]
*BICD2*^*GFP-N*^ on FVB background	*SOD1*^*G93A*^ low-copy transgenic on FVB/N background	14% increase in lifespan (271 days, *n* = 12, versus 237 days, *n* = 12	[48,92]
*Dync1h1*^*+/Loa*^ on C57BL/6 congenic background	*SOD1*^*G37R*^ transgenic on C57BL/6 congenic background	No significant differences in lifespan (192 days, *n* = 10, compared with 189 days, *n* = 28)	[34]
*Dync1h1*^*+/Loa*^ on C57BL/6 congenic background	*SOD1*^*G85R*^ transgenic on C57BL/6 congenic background	6% increase in lifespan (386 days, *n* = 14, compared with 364 days, *n* = 43)	[34]

a Effects on lifespan (to humane end point) of double-mutant progeny from crossing SOD1-ALS mutant mice to animals with mutations in *Dync1h1* or other strains discussed in this review.

## Box 1 Dync1h1Loa, Dync1h1Cra1, and Dync1h1Swl mutations and the Harwell connection

In 1994, one of the authors was talking to a member of Professor J. Peters’ Laboratory of the MRC Mammalian Genetics Unit in Harwell, UK, a world centre for mouse genetics since 1946. The Peters laboratory produced novel mouse mutants with the chemical mutagen *N*-ethyl-*N*-nitrosourea (ENU), which typically causes point mutations, particularly A-to-T changes [95]. A discussion arose about a new mutation that had disrupted locomotion. The first mouse described was an obligate heterozygote female born in 1984 from a C3H/HeH male previously treated with ENU and crossed to a PTP-strain female. This ‘Legs at odd angles’ (*Loa*) mouse was noticed because of her unusual locomotion and named because she held her legs at a strange angle (limb grasping, a generic sign of neuromuscular/motor system deficits) when rapidly suspended by the tail. This mouse and her progeny were crossed to several inbred strains, but mainly the 101 strain. During the following behavioural characterisation [96], it was noticed that heterozygous animals, which otherwise lived to a normal age compared with wild type littermates, developed a slowly progressive locomotor deficit that manifested as a low-based ‘reptilian’ gait and Rotarod test and grip-strength defects.

In that pre-genome project era, positional cloning of an unknown mutant gene was a gruelling undertaking of several years. Having found linkage of the *Loa* trait to mouse chromosome 12, the physical maps spanning the critical region were published [97]. In 2001, the critical region was displayed at the 15th International Mouse Genome Conference, Edinburgh, UK. Dr Andreas Russ, then working for the German Biotech company Ingenium saw the map and realised that the description of the mutant and the position of the unknown mutation were almost identical to that of a mouse mutation, Cramping 1, created by the German ENU project [98]. The groups in the UK and Germany cooperated and in 2003 found that the *Loa* and *Cra1* strains have point mutations in *Dync1h1*. The Loa mutation is a transversion resulting in a change from phenylalanine to tyrosine at position 580 (F580Y), whereas the *Cra1* mutation changes tyrosine to cysteine at position 1055 (Y1055C) [15]. The non-complementation found in the compound heterozygotes (*Dync1h1*^*Loa/Cra*^*)* added considerable weight of evidence that the correct mutations had been identified.

The first Sprawling mouse, described in 1967, had been produced from an irradiation experiment [18,19]. However, the causative mutation was published only in 2007 by Popko and colleagues, who determined that the phenotype arose from a 9-bp deletion in exon 12 of *Dync1h1* that changed four amino acids (GIVT) to a single alanine (A) residue in a cargo-binding domain [14]. The Sprawling mouse strain originated at MRC Harwell.

## Box 2 Outstanding questions

• How do mutations in *Dync1h1* and *DYNC1H1* generate such a broad spectrum of phenotypes ranging from motor neuropathies to malformations of the cerebral cortex?
• Why do these mutations have cell specificity?
• How many different types of cytoplasmic dynein complex exist in a eukaryotic cell?
• How is the assembly of cytoplasmic dynein complexes of specific composition regulated in different cell types?
• What is the basis of the functional interaction between *SOD1* and *Dync1h1* in mice and humans?
• Why do the known *Dync1h1* and *DYNC1H1* mutations have overlapping phenotypes but remain distinct from each other?
• Why do nearly overlapping mutations in *Dync1h1* and *DYNC1H1* give rise to dramatically different phenotypes and clinical outcomes?
• What is the precise role of genetic background in the clinical phenotypes of *Dync1h1* and *DYNC1H1* mutations?
• What are the roles of the novel domains identified by the mutations recently found in DYNC1H1 in dynein function and regulation?
